# The Common Task

**Published:** 1907-06-15

**Authors:** 


### Nursing Administration.

### THE COMMON TASK.

### Beef Tea for Institutional Use.

It is a very difficult point to decide what amount of nourishment is imparted to the patient through the medium of ordinary beef tea. The various attempts to prepare analyses have exposed the extraordinary variations which occur in these preparations according to the materials used and the mode of infusing the tea. All that is certain is this: no form of beef tea, whether home-made or manufactured in a concentrated form, is found to be satisfactory as the sole constituent in the diet. It is a useful, in some cases it would seem an indispensable, addition to the diet, but cannot be depended on for long to nourish the patient without milk or some kind of solid food. Its use in hospitals and infirmaries has become a matter of habit and routine, to an [illegible]ent which is likely to be greatly modified as [illegible]r ideas of economical housekeeping make [illegible]mselves felt. For it is the essence of "beef tea" in its strict form that it should contain nothing but pure extract of beef, and this altogether precludes in the eyes of the ordinary housekeeper the admixture of the many appetising fragments which should find a place in the stock pot, and which, when duly strained and skimmed, afford excellent broth at no cost to the hospital whatever. We have shown the cost of some well-known concentrated preparations of beef tea contrasted with that of the home-made variety. But cost is not the only factor to be considered. In a matter affecting invalids, flavour is all important, and it is essential that a guarantee should be given that certain necessary properties are present in due proportion. This most well-known manufacturers are willing to give. We have examined all the leading brands of beef tea with a view to their use in institutions, and have reached the following conclusions:—

That the more closely the question of beef tea is considered, the more clearly it is perceived that its preparation tends to fall into the category of skilled processes, demanding technical training and special apparatus.

As regards food value, we are much disposed to believe that, taking into consideration the rough-and-ready way in which beef tea is commonly prepared in the institution, a method through which a good proportion of the value of the tea is thrown away among the rags of beef, the advantage is likely to be on the side of the manufactured article, for the processes of extracting the essence of meat have been carefully thought out by the makers, and are intelligently supervised. It is on the face of it less likely that large losses in the qualities of the meat should be allowed to occur in factories devoted to the one aim of obtaining the best results than in the hurried atmosphere of the hospital kitchen.

Without going to the length of the writer who declares "all the bloodshed caused by the warlike ambition of Napoleon is as nothing compared with the myriads of persons who have sunk into their graves from misplaced confidence in the food value of beef tea," we may confidently assert that thousands of pounds' worth of good nourishment are annually thrown away in institutions for the sick, owing to wasteful methods of preparing this time-honoured article of diet. It is interesting in this connection to recall the result of an analysis made at the requisition of Mr. Sydney Phillips, Steward of St. Thomas's Hospital, and printed in The Hospital, June 11, 1904:—"A surprise visit was made to the kitchen one morning, and a quart sample of the beef tea was taken and put into a bottle labelled A." This was then analysed with the following result: Water, 96.03; fat, 0.199; insoluble proteids and meat fibre, 0.208; soluble proteids and gelatine, 1.342; meat bases, 0.608; non-nitrogenous extractive matters, 0.843; mineral matter, 0.77: total, 100. Total dry solid matter, 3.970 per cent.

The chemist proceeds to remark that "it must be borne in mind that such preparations have extremely small, if any, value as real foods. They must be regarded as stimulants and restoratives, and this only in a limited sense, owing to the large proportion of water they contain. It will be noticed that the total dry solid matter amounts to only 3.970 per cent. in A. Deducting the sum of the meat bases, extractives and salts (valuable merely as stimulants), together with the proportion of fat and also disregarding the vexed question as to the real nutritive power of gelatine, there is left to A 1.55 per cent. nitrogenous organic matter." An analysis made at the same time of a "well known meat extract," not otherwise specified, revealed an even smaller proportion of "nitrogenous organic matter," namely, 0.879. Now, taking into consideration that each patient gets not a quart, but half a pint of this mixture, and that the amount of "nitrogenous organic matter" which he thereby imbibes is infinitesimal, it may be asked for what purpose the hospital elects to expend so much labour and money in the process of beef tea making. If this form of nourishment i[illegible]ndispensable, as is commonly believed, it is well worth while for institutions to invite samples of different preparations, and decide, after making their own analyses, which process out of the many advertised, whose names we have placed before our readers, is most successful in presenting a concentrated beef tea, in a fluid, or easily soluble form. Here, as elsewhere in hospital economy, it is not always cheapness which wins the day. The really important thing is get the best value for the money. There is little doubt that among the grave sources of waste in institutions for the sick must be reckoned the annual pouring down the throats of the patients countless gallons of relatively worthless, and certainly very costly beef tea.

